# Arbuscular Mycorrhizal Fungi for the Biocontrol of Plant-Parasitic Nematodes: A Review of the Mechanisms Involved

**DOI:** 10.3389/fmicb.2015.01280

**Published:** 2015-11-17

**Authors:** Nele Schouteden, Dirk De Waele, Bart Panis, Christine M. Vos

**Affiliations:** ^1^Laboratory of Tropical Crop Improvement, Department of Biosystems, KU Leuven, Heverlee, Belgium; ^2^Unit for Environmental Sciences and Management, North-West University, Potchefstroom, South Africa; ^3^Bioversity International, Heverlee, Belgium; ^4^Centre of Microbial and Plant Genetics, KU Leuven, Heverlee, Belgium; ^5^Department of Plant Systems Biology, Vlaams Instituut voor Biotechnologie, Gent, Belgium; ^6^Commonwealth Scientific and Industrial Research Organisation Agriculture, Queensland Bioscience Precinct, Brisbane, QLD, Australia

**Keywords:** arbuscular mycorrhizal fungi, biocontrol, cyst nematodes, induced systemic resistance, plant-parasitic nematodes, migratory nematodes, mycorrhiza induced resistance, root-knot nematodes

## Abstract

Arbuscular mycorrhizal fungi (AMF) are obligate root symbionts that can protect their host plant against biotic stress factors such as plant-parasitic nematode (PPN) infection. PPN consist of a wide range of species with different life styles that can cause major damage in many important crops worldwide. Various mechanisms have been proposed to play a role in the biocontrol effect of AMF against PPN. This review presents an overview of the different mechanisms that have been proposed, and discusses into more detail the plausibility of their involvement in the biocontrol against PPN specifically. The proposed mechanisms include enhanced plant tolerance, direct competition for nutrients and space, induced systemic resistance (ISR) and altered rhizosphere interactions. Recent studies have emphasized the importance of ISR in biocontrol and are increasingly placing rhizosphere effects on the foreground as well, both of which will be the focal point of this review. Though AMF are not yet widely used in conventional agriculture, recent data help to develop a better insight into the modes of action, which will eventually lead toward future field applications of AMF against PPN. The scientific community has entered an exciting era that provides the tools to actually unravel the underlying molecular mechanisms, making this a timely opportunity for a review of our current knowledge and the challenges ahead.

## Introduction

Nematodes form a highly diverse group comprising free-living nematodes as well as plant and animal parasites that can be found worldwide in various habitats (Ferraz and Brown, 2002). Many species of plant-parasitic nematodes (PPN) can act as pests on a wide range of important agricultural crops. They mostly live in the soil, but some species such as several *Ditylenchus* spp. can act as aboveground pests. PPN show a wide array of life styles, but all have a usually hollow, retractable, needle-like mouth spear called the stylet for feeding. They are classified into different groups based on their feeding strategy (Perry and Moens, 2011). Ectoparasitic nematodes remain in the rhizosphere during feeding, using their stylet to acquire food from the epidermal or outer root cortex cells. Endoparasitic nematodes on the other hand completely enter the root and feed inside the root. Migratory endoparasitic nematodes (e.g., *Radopholus* spp. and *Pratylenchus* spp.) migrate inter-or intracellularly while feeding on root cortex cells, thus causing damage to the plant along their migration path (Jones et al., 2013). Endoparasitic sedentary nematodes display the most complex feeding strategy of PPN, selecting cells in the vascular cylinder to be converted into a feeding site and then becoming sedentary with the onset of feeding (Gheysen and Mitchum, 2011). This last group includes the cyst and root-knot nematodes, which are considered to be the most damaging pests of agricultural crops worldwide (Jones et al., 2013; Bartlem et al., 2014). The sedentary endoparasitic *Meloidogyne* spp. such as *M. incognita* and *M. javanica*, can result in complete crop losses in tobacco and tomato or sunflower and pepper, respectively (Wesemael et al., 2011). The direct damage caused by PPN can be aggravated by secondary infections of the wounded plant tissues by other pathogens, while some PPN, such as the migratory ectoparasitic *Xiphinema* spp., can transmit plant viruses (Hao et al., 2012). Yield losses caused by PPN are expected to rise in the near future as a result of climate change and cropping systems intensification (Nicol et al., 2011). The use of nematicides is being limited, given the increasing concern for human health as well as the environment, which has led to their ban. Alternative nematicides are being sought (Oka and Mizukubo, 2009; Wesemael et al., 2011). Scientists are also looking for other nematode management strategies that fit into the recently launched framework of the Integrated Pest Management (IPM) directive of the European Union (EU directive 2009/128/EC), stating that member states have to implement IPM from 2014 onward, with the aim to reduce pesticide use and to promote non-chemical management practices as much as possible. One of the proposed environmentally friendly options to manage PPN is the use of biological control organisms, such as arbuscular mycorrhizal fungi (AMF).

Arbuscular mycorrhizal fungi are obligate root symbionts, estimated to colonize more than 80% of all land plant species. They improve plant growth through increased nutrient uptake in exchange for photosynthetic carbon from their host (Smith et al., 2010). Also, they can alleviate plant stress caused by abiotic as well as biotic factors, including PPN (Gianinazzi et al., 2010; Singh et al., 2011; Vos et al., 2012a). The biocontrol effect of AMF has been observed in a wide range of plant species and against many pathogens, most of them soil-borne fungal pathogens causing root rot or wilting, though successful biocontrol has also been observed against aboveground pathogens such as *Alternaria solani* in tomato (Harrier and Watson, 2004; Whipps, 2004; Fritz et al., 2006; Pozo and Azcón-Aguilar, 2007; Jung et al., 2012). Both necrotrophic and biotrophic pathogens have been reported to be suppressed by AMF, either directly or indirectly (Veresoglou and Rillig, 2012). AMF can also suppress PPN, as has been previously reviewed by Pinochet et al. (1996) and Hol and Cook (2005). *In vitro*, greenhouse as well as field experiments indicated protective effects against PPN by AMF in plants such as banana, coffee and tomato (Calvet et al., 2001; Vos et al., 2012b; Alban et al., 2013; Koffi et al., 2013). These protective effects ranged from a reduction in infection and reproduction to an enhanced tolerance. But though there are many reports on the biocontrol effect of AMF, their actual use as biological control agents in the field is still not a routine agricultural practice (Salvioli and Bonfante, 2013). This is partially due to variability in performance, depending on the AMF isolate, pathogen, plant species and environmental conditions (Dong and Zhang, 2006; Veresoglou and Rillig, 2012; Salvioli and Bonfante, 2013). An increased insight into their modes of action will therefore help to increase the efficacy of these biocontrol agents.

**FIGURE 1:**
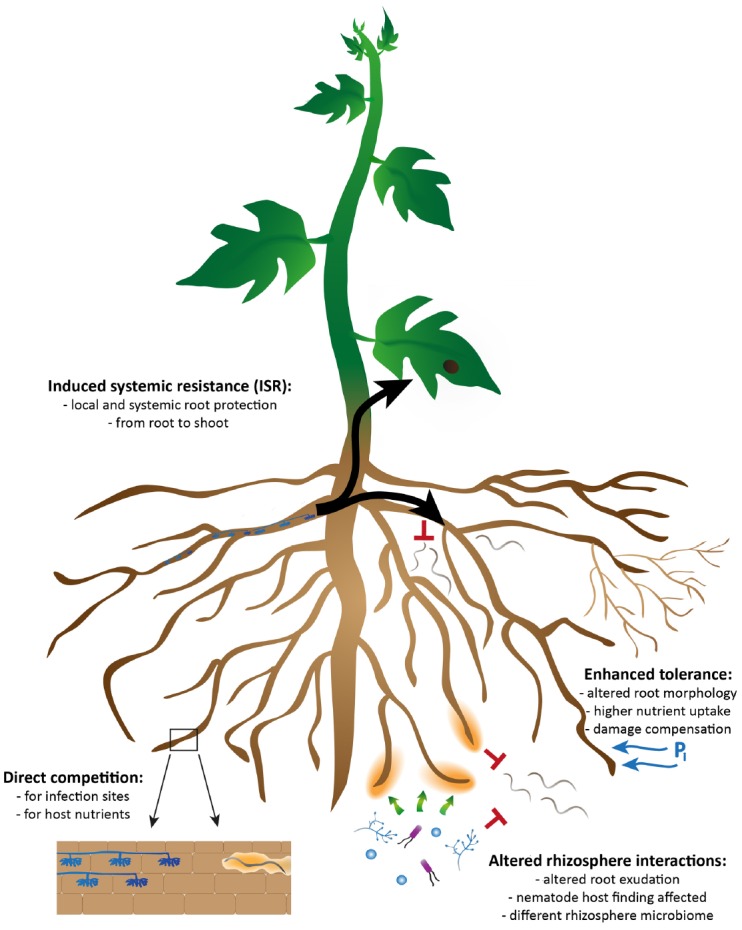
**Overview of the possible mechanisms by which arbuscular mycorrhizal fungi can exert biocontrol against plant-parasitic nematodes.** They consist of direct effects of AMF on the pathogen, involving competition for space and nutrients (bottom left) and indirect plant-mediated effects, involving damage compensation and enhanced tolerance (top right). The latter can be further divided into the effects of AMF on plant defense induction (ISR; top left) and altered rhizosphere interactions through changes in plant root exudation (bottom right). The different mechanisms cannot be considered as completely independent from each other and biocontrol probably results from a combination of different mechanisms.

Several mechanisms can be involved in the AMF-mediated biocontrol; direct effects of AMF on the pathogen, involving competition for space or nutrients, or indirect, plant-mediated, effects. The latter can further be divided into the effects of AMF on plant tolerance, plant defense induction and altered plant exudation leading to altered rhizosphere interactions (Figure 1). The different mechanisms cannot be considered as completely independent from each other and biocontrol probably results from a combination of different mechanisms (Vierheilig et al., 2008; Cameron et al., 2013). In addition, the relative importance of a specific mechanism can vary depending on the specific AMF-pathogen-plant interaction. In recent years, much progress has been made, especially in the domains of induced systemic resistance (ISR; Pieterse et al., 2014) as well as on the role of the rhizosphere in biological control (Cameron et al., 2013), which will be the focal points of this review. We will present an overview of the different mechanisms that have been proposed to play a role in the AMF-mediated biocontrol, and we will discuss more into detail the possible significance of the different mechanisms for plant infection by PPN.

## Enhanced Plant Tolerance

### Higher Nutrient Uptake

Arbuscular mycorrhizal fungi are known to be able to increase the uptake of water and mineral nutrients for their host plant, such as phosphate and nitrogen (Parniske, 2008; Baum et al., 2015) but probably also micro-elements such as zinc (Smith and Smith, 2011a,b). In return, they receive photosynthetic carbon from their host (Gianinazzi et al., 2010). Similar to the protection of the plant by AMF against various abiotic stress factors such as drought, cold or heavy metal toxicity (Singh et al., 2011), AMF could also compensate for damage caused by pathogens. Although higher uptake of phosphate has been proposed as a mechanism for the AMF-mediated biocontrol, addition of phosphate to non-mycorrhizal plants did not result in a similar reduction of pathogen infection (Bodker et al., 1998). Fritz et al. (2006) showed that tomato plants colonized by *Rhizophagus irregularis* showed significantly less symptoms caused by *A. solani* than non-mycorrhizal plants, while no increase in phosphate uptake was observed. An additional phosphate supply even resulted in higher disease incidence. There is thus not always a positive correlation between increased phosphate uptake and plant growth promotion in mycorrhizal plants, as in some cases plant growth suppression resulted as a consequence of AMF colonization, even when phosphate transport from the AMF to the host plant was taking place (Smith and Smith, 2011a).

Plants with a better nutrient status are able to tolerate higher PPN population densities in their roots, as observed in cotton fields infested with the sedentary semi-endoparasitic nematode *Rotylenchulus reniformis* (Pettigrew et al., 2005). Regression analysis of nematode population densities against the mineral content in rice also revealed a positive correlation between migratory ectoparasitic *Helicotylenchus* spp. and Mg, however, a negative correlation was observed between the migratory endoparisitic nematode *Pratylenchus zeae* and Zn or Fe, and between *M. incognita* and Mg and Ca (Coyne et al., 2004). These observations indicate that the nutrient status of the host plant can affect PPN population densities in both a positive and negative way. But so far, no solid data are available that prove that the AMF-enhanced nutrient status is a causal agent of a higher resistance against PPN.

### Altered Root Morphology

Apart from an increased nutrient status, mycorrhizal plants often show increased root growth and branching (Gamalero et al., 2010; Orfanoudakis et al., 2010; Gutjahr and Paszkowski, 2013). The root morphology responses resulting from AMF colonization seem to depend on plant characteristics, with tap roots for example appearing to profit more from AMF than fibrous roots in terms of gained biomass and nutrient acquisition (Yang et al., 2014).

Increased root branching observed in mycorrhizal plants has been suggested to have implications for pathogen infection as well (Vos et al., 2014), although a clear correlation could not been found (Vierheilig et al., 2008). Positive effects could result from an increase in root vigor, due to a higher nutrient uptake capacity. It might even counterbalance the suppressed root growth caused by PPN. For example, decreased root branching caused by the migratory endoparasitic nematodes *Radopholus similis* and *P. coffeae* in banana was counterbalanced by the increased branching due to colonization by the AMF *Funneliformis mosseae* (Elsen et al., 2003a). Increased root branching can, however, also have a negative impact on the host plant by an increase in potential infection sites, depending on the PPN and plant species. Migratory endoparasitic nematodes, such as *R. similis*, have a preference for primary roots (Stoffelen et al., 2000; Elsen et al., 2003b). For the sedentary endoparasitic root-knot and cyst nematodes, the root elongation zones and sites of lateral root formation are preferred penetration sites, probably because of increased leakage of exudates in these zones (Wyss, 2002; Curtis et al., 2009). However, increased root branching and root length did not alter plant susceptibility to cyst nematodes, as it was observed that the number of penetrating *Heterodera schachtii* juveniles in transgenic *Arabidopsis thaliana* plants with long- or short-root phenotypes was similar to that in wild type plants (Hewezi and Baum, 2012).

The possible role of altered root morphology in AMF-mediated biocontrol has been investigated against several other pathogens. In an earlier study, Norman et al. (1996) investigated the consequences of a highly branched root system of strawberry on *Phytophthora fragariae* infection, a pathogen that mainly infects through the root tips. For non-mycorrhizal root systems infection was indeed higher on the more highly branched roots, but this was not observed in mycorrhizal root systems. Similar findings in different AMF-pathosystems were also reported later (Fusconi et al., 1999; Gange, 2000; Vigo et al., 2000; Gamalero et al., 2010), pointing toward other mechanisms involved in the AMF-mediated biocontrol. Although such experiments have not been performed specifically with PPN, similar mechanisms can be expected to play a role as well.

## Direct Competition for Nutrients and Space

Although no antibiotic production or mycoparasitism potential has so far been shown in AMF species, direct effects of AMF on pathogen infection through competition have been proposed. Competition for nutrients or for space and infection sites do occur between micro-organisms with the same physiological requirements in an ecological niche, especially where resources such as carbon might be limited (Vos et al., 2014).

Nutrient competition, with emphasis on competition for carbon, has been suggested as a mechanism of the AMF-mediated biocontrol though not much evidence is found in the literature (Jung et al., 2012). The carbon transfer from the host plant to the AMF is estimated to range from 4 to 20% of the total assimilated carbon (Hammer et al., 2011). It thus seems plausible that AMF compete with pathogens for this resource (Vos et al., 2014). As there is a difference in carbon sink strength between different AMF species, according to the hypothesis of carbon competition it could thus be expected that different AMF species mediate different levels of biocontrol (Lerat et al., 2003). Thus far, however, this hypothesis is not supported by experimental evidence (Vierheilig et al., 2008; Jung et al., 2012). For example, the AMF *R. irregularis* could not exert a stronger biocontrol effect on *R. similis* and *P. coffeae* in banana nor on *M. incognita* in tomato despite its higher carbon sink strength compared to *F. mosseae* (Vos, 2012).

**FIGURE 2:**
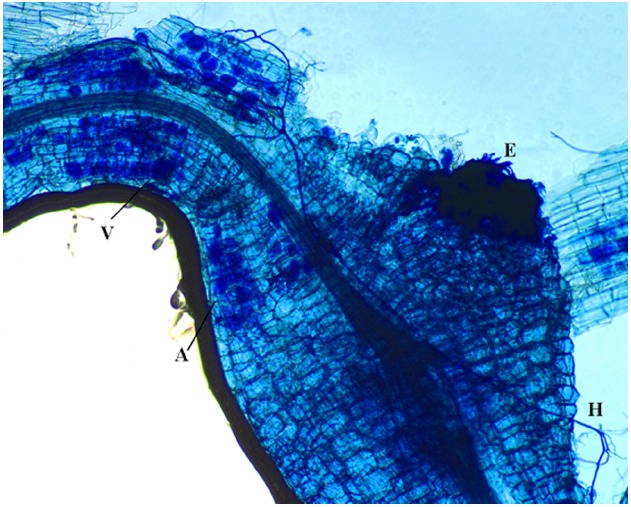
**Tomato roots stained with ink-vinegar solution to observe mycorrhizal colonization by ***Funneliformis mosseae*** (in blue).** This root fragment shows typical mycorrhizal structures such as hyphae (H), arbuscules (A) and vesicules (V), showing the mature stage of the colonization along with the gall induced by *Meloidogyne incognita*, of which the egg mass (E) is also visible. As such, direct competition for space between arbuscular mycorrhizal fungi and sedentary endoparasitic nematodes probably can take place. Bar size = 100 μm.

Competition for space could also be involved in AMF-PPN interactions since they both reside in roots (Jung et al., 2012). Negative effects due to space constriction can be exerted on PPN as mycorrhizal arbuscules exclusively form in the cortex, where also migratory endoparasitic nematodes feed. Space competition between AMF and sedentary endoparasitic nematodes could be brought into play in case the feeding cells extend into the cortex (Figure 2). Cyst nematode feeding cells, the so-called syncytia, are confined within the endodermis and should therefore be less affected by AMF. Through PCR and DNA sequencing, del Mar Alguacil et al. (2011) reported that galls produced by *M. incognita* in *Prunus persica* roots could be colonized by AMF. However, as mycorrhizal arbuscules are short-lived structures (Parniske, 2008; Javot et al., 2011), it is difficult to distinguish whether AMF or PPN colonized the same root part first.

Competition for space also implies that a higher AMF colonization degree of the root should lead to a higher level of AMF-mediated biocontrol (Vierheilig et al., 2008). This hypothesis, however, holds only true to a certain degree. A mature AMF colonization, characterized by the presence of arbuscules, seems to be a prerequisite for biocontrol (Khaosaad et al., 2007; Pozo and Azcón-Aguilar, 2007). Dos Anjos et al. (2010) also concluded that when the symbiosis was well established prior to *M. incognita* inoculation, *M. incognita* reproduction was reduced, whereas co-inoculation had no effect. However, when co-inoculating native AMF together with *Meloidogyne exigua* in coffee plants, a biocontrol effect was observed (Alban et al., 2013). The competition for nutrients and space also implies that AMF could be affected by PPN infection as well. Hol and Cook (2005) concluded following a meta-analysis of the available literature that AMF colonization was reduced by ectoparasitic, migratory endoparasitic and sedentary endoparasitic nematodes. In greenhouse experiments, *R. similis* and *P. coffeae* in banana affected the frequency of *F. mosseae* colonization, but not the intensity (Elsen et al., 2003a,b). Contrastingly, root colonization by *R. irregularis* in *in vitro* banana plantlets was not affected either by *R. similis* (Koffi et al., 2013) or by *P. coffeae* in transformed carrot roots (Elsen et al., 2003c). For sedentary endoparasitic nematodes, Dos Anjos et al. (2010) showed that *M. incognita* could negatively affect the sporulation of the AMF *Scutellospora heterogama* in sweet passion fruit, while Alban et al. (2013) found that pre-inoculation of *M. exigua* led to a significant increase in the subsequent colonization of AMF compared to uninoculated mycorrhizal plants. del Mar Alguacil et al. (2011) also reported that the highest AMF diversity was found in uninfected roots compared to *M. incognita* infected roots and galls, and that the composition of the AMF community was different between infected and uninfected roots. Their results indicate that AMF colonization might also be suppressed by PPN, depending on the AMF species as some AMF species were not affected by the PPN.

## Effects Through Induced Systemic Resistance

In many cases the above mentioned mechanisms are not able to explain the observed AMF-mediated biocontrol, as plant-mediated responses seem also to be involved. For example, using a split-root experimental set-up in tomato in which the root system of one plant was divided over two physically separated compartments with one side pre-inoculated with *F. mosseae*, Vos et al. (2012b) demonstrated that *M. incognita* or *P. penetrans* infection in the other root compartment was significantly reduced. De la Peña et al. (2003), however, did not find a systemic biocontrol effect on *P. penetrans* in the dunegrass *Ammophila arenaria* pre-colonized by different native AMF species. However, more reports highlight a systemic suppression of nematode infection in mycorrhizal roots. As, for example, systemic suppression was observed in split root experimental set-ups in *R. irregularis*-colonized banana against *R. similis* and *P. coffeae* (Elsen et al., 2008) but also in grapevine against the ectoparasitic nematode *X. index* (Hao et al., 2012). A systemic biocontrol effect of AMF has also been shown in interactions with several other pathogens (Cordier et al., 1998; Pozo et al., 2002; Zhu and Yao, 2004; Fritz et al., 2006; Khaosaad et al., 2007; Castellanos-Morales et al., 2011), supporting the importance of this mechanism in the AMF-mediated biocontrol.

Novel insights that are shaping our understanding of the induction of systemic plant defense responses by AMF include the notion that plants initially perceive beneficial organisms as putative pathogens, due to MAMPs (microbe-associated molecular patterns) being conserved between beneficial and pathogenic fungi (Zamioudis and Pieterse, 2012). With AMF being obligate biotrophs, it has indeed been shown that a significant overlap exists in the transcriptional profile of the plant response to AMF and a biotrophic pathogen, such as *Magnaporthe grisea* (Paszkowski, 2006). Then, upon MAMP-recognition by the plant’s pattern recognition receptors, a MAMP-triggered immunity response (MTI) is activated, forming the first line of defense of the plant in an effort to limit further pathogen invasion (Jones and Dangl, 2006). Surprisingly, the presence of an MTI response in the plant roots has only recently been demonstrated (Millet et al., 2010).

Being initially perceived by the plant as a putative biotrophic pathogen, AMF thus also induce a MTI response, leading to transcriptional and hormonal changes in their host plant upon establishment of the symbiosis. Fiorilli et al. (2011) studied the transcriptome of tomato plants during the colonization process by *F. mosseae* and observed significant gene modulation in both roots and shoots, with the largest alterations in primary and secondary metabolism, as well as in defense and response to biotic stimuli. López-Ráez et al. (2010) compared the transcriptional response of tomato to two AMF differing in colonization pattern, namely *F. mosseae* and *R. irregularis*. Despite the common induction of jasmonate (JA)-biosynthesis and signaling-related genes, they only found 35% overlap in the overall transcriptional profiles of tomato roots colonized by either of these two AMF species. In the case of *F. mosseae*, a stronger induction of the largely root-specific 9-lipoxygenase (9-LOX) pathway was observed, as well as the induction of the isoleucine conjugate of JA (JA-Ile), several JA-dependent markers and increased salicylic acid (SA) levels, which could be linked to its lower degree of colonization compared to *R. irregularis*. The early MTI-response involving the jasmonate-linked 9-LOX-pathway could have an early effect on root-knot nematodes as well. In maize, the expression or presence of the 9-LOX gene (*ZmLox3*) proved to be essential for resistance against *M. incognita* (Gao et al., 2008). However, so far tripartite studies involving the plant, AMF and PPN have not reported similar results.

Typically, the induction of an MTI response in the early stages of AMF colonization is only weak and transient, so that a further successful establishment of the symbiosis becomes possible. AMF try to avoid their detection by the plant as much as possible, but also actively suppress the MTI response using effectors (Zamioudis and Pieterse, 2012). As a biotroph, AMF colonization is negatively impacted by SA, which is why they are thought to attempt to suppress the SA-mediated defense response in the plant (Hause et al., 2007; Miransari et al., 2014). In addition, it has been proposed that the establishment of the symbiotic program, activated upon perception of mycorrhizal Myc factors, also counteracts the MTI response (Zamioudis and Pieterse, 2012). So far, only one effector molecule has been described for AMF, being the SP7 effector of *R. irregularis* that interferes with ethylene (ET) signaling in the plant (Kloppholz et al., 2011). This can be associated with recent reports highlighting the role of ET in the MTI response (Millet et al., 2010).

The initial interaction phase between AMF and its host plant primes the plant for a faster and stronger induction of usually JA-dependent defense responses upon subsequent pathogen attack. This cost-effective phenomenon is described as ISR, and specifically in the case of AMF the term mycorrhiza-induced resistance (MIR) has been proposed (Pozo and Azcón-Aguilar, 2007; Pieterse et al., 2014). Exogenous JA-applications and study of mutants altered in the JA-pathway have proven that the JA-dependent pathway is indeed able to mediate resistance to PPN (Soriano et al., 2004; Cooper et al., 2005; Fujimoto et al., 2011; Fan et al., 2015). Traditionally, defense responses to microbes fall into two categories, being termed either ISR or systemic acquired resistance (SAR). SAR is typically SA-dependent and leads to the induction of pathogenesis-related (PR) proteins, while ISR is defined as being regulated by JA and ET, and not accompanied by major changes in PR protein expression (Vlot et al., 2008; Pieterse et al., 2009). Recent research efforts, however, prove that the overlap between SAR and ISR is much larger than originally thought, with substantial crosstalk taking place between the different pathways (Mathys et al., 2012; Pieterse et al., 2014). In this context, the MIR defense response against PPN is probably not solely linked to the JA-dependent pathway.

Some light has been shed on this topic by a few transcriptome studies performed in recent years, involving the tripartite interaction of plants, AMF and nematodes. Li et al. (2006) reported the primed transcriptional activation of a class III chitinase gene in *Glomus versiforme* colonized grapevine roots upon infection by *M. incognita*. Constitutive expression of this gene in transgenic tobacco plants enhanced the resistance against the RKN, but did not affect the AMF. This strongly suggests that the class III chitinase gene is involved in a protective mechanism against the PPN. Other recent studies also show that transgenic plants with higher chitinase activity were more resistant to RKN. Though this mostly affected the viability of the eggs which contain chitin, it also reduced the amount of egg masses and thus productivity of the females (Chan et al., 2010, 2015). The primed activation of several other plant defense-related genes was recently also reported in *R. irregularis* colonized grapevine (*Vitis* spp.) after infection by the ectoparasitic *X. index* (Hao et al., 2012). Expression analyses of expressed sequence tags (ESTs) generated by suppression subtractive hybridization (SSH) showed several plant genes that are upregulated during MIR. Interestingly, these genes were only upregulated when AMF and nematode were both present in the root, indicating a priming of these defense genes. The products of these genes include chitinase 1b, but also PR 10, which has RNase and antimicrobial activity. Interestingly, a PR10 protein purified from *Crotalaria pallida* shows nematostatic and nematicidal effects against *M. incognita*, targeting a digestive proteinase of the nematode (Andrade et al., 2010). Furthermore, the SSH-study by Hao et al. (2012) showed that glutathione S-transferase was upregulated, which is probably involved in the detoxification of reactive oxygen species (ROS) that can be imposed by the stress of the cell’s hypertrophy and necrosis following nematode infection. Stilbene synthase 1 was also upregulated. It is a key enzyme in the phenylpropanoid pathway toward the phytoalexin resveratrol, of which the accumulation is a typical defense response by grapevine to biotic or abiotic stresses. However, in a previous research, resveratrol was not found to be effective *in vitro* on *R. similis*, *P. penetrans* nor *M. incognita* (Wuyts et al., 2006). Genes of 5-enolpyruvyl shikimate-3-phosphate synthase (ESPS) and a heat shock protein 70-interacting protein (HIP) were also primed (Hao et al., 2012). ESPS catalyzes the penultimate step in the shikimate pathway. The differential expression of shikimate pathway genes has been reported before in response to root-knot nematode infection of tomato (Schaff et al., 2007) and is thought to be related to the regulation of the auxin balance which is of importance for nematode feeding site formation and possibly location (Curtis, 2007; Gheysen and Mitchum, 2011).

More recently, Vos et al. (2013) found through SSH a clear primed defense response against *M. incognita* by *F. mosseae* in tomato. The identified differential expressed genes were mainly classified in the categories of defense, signal transduction and protein synthesis and modification. For example, there was a primed upregulation of chorismate synthase, which catalyzes the conversion of the ESPS product to chorismate, which is the last step in the shikimate pathway. The shikimate pathway thus seems to be implicated in AMF-mediated biocontrol in different plant species against several types of nematodes (Hao et al., 2012; Vos et al., 2013). Furthermore, the shikimate pathway produces precursors for various aromatic secondary metabolites which are produced through the phenylpropanoid pathway among which flavonol synthase has been reported to be primed (Vos et al., 2013). The detrimental role of several phenylpropanoid pathway products, including flavonols, on *M. incognita*, *R. similis*, and *P. penetrans* has already been demonstrated *in vitro* (Wuyts et al., 2006). Through SSH, also the ROS metabolism was linked to the reduction of root-knot nematode infection in mycorrhizal tomato roots (Vos et al., 2013). Similarly, Beneventi et al. (2013) suggested an important role for ROS generation in the resistance of soybean to *M. javanica* as they found through pyrosequencing an over–representation of genes containing various oxidase and peroxidase domains upregulated in the incompatible interaction. Other noteworthy genes found to be primed upon infection by *M. incognita* in mycorrhizal tomato plants by Vos et al. (2013) were sinapoylglucose:choline sinapoyltransferase, involved in the biosynthesis of a lignin precursor and 1-aminocyclopropane-1-carboxylate oxidase (ACC oxidase), catalyzing the final step in the biosynthesis of ethylene and related to the jasmonate-ethylene dependency of ISR (Verhagen et al., 2004; Schäfer et al., 2009). These transcriptome data point toward several candidate genes by which AMF can exert biocontrol against PPN through priming of effective plant defenses. However, future research should focus on the elucidation of their specific action toward PPN, with emphasis on the proteome and metabolome AMF-associated changes.

## Altered Rhizosphere Interactions

The plant-mediated effects of the AMF-mediated biocontrol go beyond MIR though: the AMF symbiosis also leads to an altered root exudation composition and level (Hodge, 2000; Jones et al., 2004), which can in turn impact the PPN in the rhizosphere in terms of hatching, motility, chemotaxis, and host location. Differences in root exudate quantity and quality between mycorrhizal and non-mycorrhizal plants have been reported for various compounds, including sugars and organic acids (Sood, 2003; Lioussanne et al., 2008; Hage-Ahmed et al., 2013), amino acids (Harrier and Watson, 2004), phenolic compounds (McArthur and Knowles, 1992), flavonoids (Steinkellner et al., 2007) and even for the plant hormone strigolactone (López-Ráez et al., 2011). The root exudation furthermore depends on the plant or AMF species involved (Badri and Vivanco, 2009; Kobra et al., 2009), as well as on the degree of symbiosis (Scheffknecht et al., 2006; Lioussanne et al., 2008).

**FIGURE 3:**
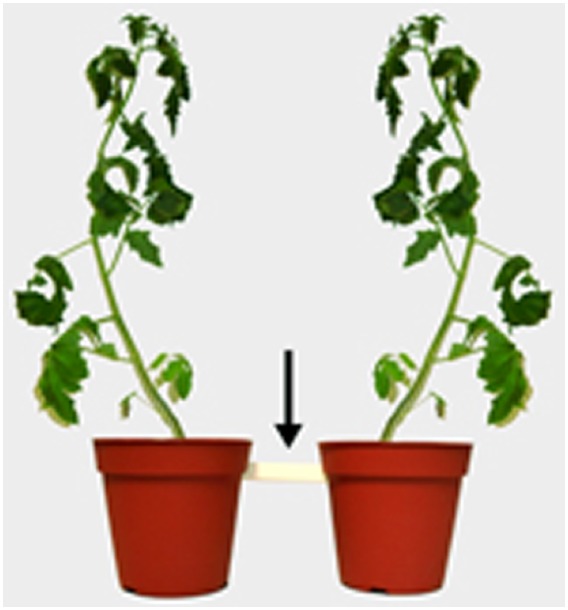
**Visualization of the experimental set-up of a twin-chamber with tomato plants used by **Dababat and Sikora (2007)** and **Vos et al. (2012b)** to study the differential attraction of ***Meloidogyne incognita*** to mycorrhizal roots.** The two plant compartments are connected by a bridge. The experiment consisted of three treatments: (i) control plants in both compartments (control treatment), (ii) mycorrhizal plants in both compartments (AMF treatment) and (iii) a control plant in the left compartment and a mycorrhizal plant in the right compartment (mixed treatment). Two days after transplanting, the compartments and bridge were watered to field capacity at time of inoculation and 2,000 freshly hatched *M. incognita* 2nd-stage juveniles were inoculated per twin chamber, exactly in the middle of the bridge. Twelve days after inoculation, nematode penetration was assessed in control and mycorrhizal root systems of all treatments.

**FIGURE 4:**
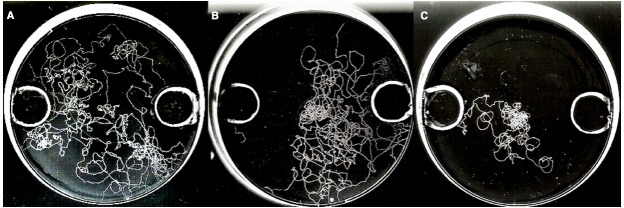
*****In vitro*** chemotaxis bio-assay with visualization of nematode tracks on the medium.** Pictures were taken 1 h after inoculation with 10 female *Radopholus similis* nematodes in the middle of the plate. Three hours before nematode inoculation, the opposite wells in the agar medium were filled with **(A)** water as control, **(B)** 1% acetic acid (left hole) *versus* 0,5M CaCl_2_ (right hole) as repellent and attractive control, or **(C)** exudates of non-mycorrhizal roots (left) *versus* exudates of mycorrhizal roots (right) to observe chemotactic behavior of the nematode. Lower nematode movement can be observed in the plate with mycorrhizal root exudates, and the nematodes clearly show a chemotactic response away from them. For experimental set-up, see Vos et al. (2012c).

Differential root exudation is an important instrument used by the host plant for autoregulation of the symbiosis (Pinior et al., 1999; Vierheilig et al., 2003; Vierheilig, 2004; Schaarschmidt et al., 2013). This phenomenon involves a differential root exudation depending upon the degree of colonization, and it has been proposed that the plant in doing so keep AMF colonization under a certain threshold, as well as deter pathogenic rhizospheric micro-organisms (Vierheilig et al., 2008). Vierheilig et al. (2000) first observed autoregulation of the AMF colonization in barley roots, and they hypothesized later that the AMF-mediated biocontrol was related to the autoregulation of the AMF symbiosis (Vierheilig et al., 2008). This hypothesis is supported by the findings of Pozo and Azcón-Aguilar (2007) that a critical degree of colonization is considered as a prerequisite for biocontrol, which is typically characterized by the presence of arbuscules. These structures are typically found in a mature symbiosis, and might thus coincide with the onset of the autoregulation process. Lioussanne et al. (2008) also observed that the attraction of *Phytophthora nicotianae* zoospores toward *R. irregularis* colonized root exudates changed to repellency, depending on the maturity of the AMF colonization. Vos et al. (2012b,c) investigated the effect of mycorrhizal root exudates on nematode behavior. Using a twin-chamber experimental set-up with tomato plants, *M. incognita* juveniles were inoculated onto a bridge connecting a tomato plant colonized by *F. mosseae* with a non-colonized plant (Figure 3). A few days later similar numbers of nematodes appeared to have moved from the bridge to either compartment, however, in the compartment with the mycorrhizal tomato, the nematodes appeared to have mainly gathered in the soil without penetrating the tomato roots, in contrast to the non-colonized compartment in which most nematodes had entered the plant roots. Further experiments indicated that *M. incognita* root penetration could even be further reduced by the additional application of mycorrhizal root exudates. Also a temporal paralysis of the second-stage infective juveniles (J2) in the presence of mycorrhizal tomato root exudates was observed in *in vitro* assays (Vos et al., 2012c). Moreover, mycorrhizal root exudates reduced host location and penetration by *R. similis* compared to non-mycorrhizal control banana plants (Vos et al., 2012c; Figure 4).

Altered root exudation can also cause a change in microbial diversity in the rhizosphere, and therefore affect plant-pathogen interactions (Bais et al., 2006; Lioussanne, 2010). Some reports show an increase in facultative anaerobic bacteria, fluorescent pseudomonads, *Streptomyces* species and chitinase-producing actinomycetes after AMF colonization (Marschner and Baumann, 2003; Wamberg et al., 2003; Harrier and Watson, 2004; Scheublin et al., 2010; Miransari, 2011; Nuccio et al., 2013; Philippot et al., 2013). These micro-organisms can also have antagonistic potential against PPN, either by direct effects such as by nematode-trapping or egg-parasitizing fungi, but also by induction of the plant defense (Kerry, 2000; Tian et al., 2007; Zamioudis and Pieterse, 2012). Root exudates originating from mycorrhizal plants have, for example, been reported to attract plant growth promoting bacteria such as *Pseudomonas fluorescens* (Sood, 2003) and to affect beneficial soil micro-organisms such as *Trichoderma* spp. (Filion et al., 1999; Druzhinina et al., 2011) which also have biocontrol potential against PPN (Dong and Zhang, 2006; Sikora et al., 2008). In line with this, Cameron et al. (2013) recently presented an adaptation to the model of MIR in which the induction of systemic resistance by mycorrhizosphere bacteria is emphasized. MIR has also been shown to act against several PPN in dixenic *in vitro* culture (Elsen et al., 2001; Koffi et al., 2013), clearly suggesting that the AMF-mediated biocontrol does not act only through a change in soil biota, but it might be an important additional factor contributing to the biocontrol under field conditions.

## Conclusion

In this review we provide an overview of the different mechanisms that have been proposed for AMF-mediated biocontrol, and specifically discuss their potential involvement in reducing PPN infections. The biocontrol effect depends on several factors such as the species involved, meaning that case-by-case studies will have to be carried out to result in field applications of AMF. Biocontrol effects of AMF on PPN have been reported since many years, but due to the technological progress that has been made in recent years we can now unravel the mechanisms behind. The sequencing of the genome of the root-knot nematodes *M. incognita* (Abad et al., 2008) and *M. hapla* (Opperman et al., 2008), the cyst nematode *G. pallida* (Cotton et al., 2014) and of host plants such as soybean (Schmutz et al., 2010), *Medicago truncatula* (Young et al., 2011) and several members of the *Solanaceae* family (Xu et al., 2011; Sato et al., 2012) are already contributing to increased insights. In addition, with the increase in microbiome studies, the importance of the mycorrhizosphere in the AMF-mediated biocontrol will be confirmed in the years to come. Progress will also be aided by the shift in attention to the plant root interactions with micro-organisms (De Coninck et al., 2015). This attention shift should already be taken into account in practice, as Castellanos-Morales et al. (2011) showed that older cultivars of wheat (before 1950) showed higher degrees of AMF root colonization compared to modern varieties (after 1950), also showing a higher bioprotective effect by AMF against the pathogen *Gaeumannomyces graminis*. Therefore, some consideration should be given to the trait of the ability to form symbiosis with AMF in future breeding efforts.

As the market for beneficial microbial inocula is growing steadily (Glare et al., 2012) and the development of beneficial microbial inocula for large-scale field application is moving forward quickly (Ijdo et al., 2011; Salvioli and Bonfante, 2013), field applications of AMF might be realistic. Thus far, few research about AMF-mediated biocontrol involved “omics” tools and systems biology approaches (Salvioli and Bonfante, 2013) but this will increase over time and provide more detailed insights in the complex mechanisms underlying AMF-mediated biocontrol. These insights might in turn lead to the effective application of AMF in the field.

### Conflict of Interest Statement

The authors declare that the research was conducted in the absence of any commercial or financial relationships that could be construed as a potential conflict of interest.
